# How Do Synchrony in Survival and Productivity Influence Abundance Synchrony in European Landbirds?

**DOI:** 10.1111/ele.70105

**Published:** 2025-05-13

**Authors:** Catriona A. Morrison, Jennifer A. Gill, Claire Buchan, Robert A. Robinson, Juan Arizaga, Oriol Baltà, Emanuel Baltag, Jaroslav Cepák, Pierre‐Yves Henry, Ian Henshaw, Zsolt Karcza, Petteri Lehikoinen, Ricardo Jorge Lopes, Bert Meister, Simone Pirrello, Kasper Thorup, Simon J. Butler

**Affiliations:** ^1^ School of Biological Sciences University of East Anglia Norwich UK; ^2^ British Trust for Ornithology Thetford UK; ^3^ Department of Ornithology Aranzadi Sciences Society Donostia Spain; ^4^ Catalan Ornithological Institute, Nat‐Museu de Ciències Naturals de Barcelona Barcelona Spain; ^5^ Marine Biological Research Station “Prof. Dr. Ioan Borcea” Agigea University “Alexandru Ioan Cuza” of Iași Iaşi Romania; ^6^ Bird Ringing Centre National Museum Praha Czech Republic; ^7^ Centre de Recherches sur la Biologie des Populations d'Oiseaux (CRBPO), Mécanismes Adaptatifs et évolution (MECADEV UMR 7179), Muséum National d'Histoire Naturelle, Centre National de la Recherche Scientifique Brunoy France; ^8^ Swedish Bird Ringing Centre, Department for Nature and Environmental Monitoring The Swedish Museum of Natural History Stockholm Sweden; ^9^ Hungarian Bird Ringing Centre, Birdlife Hungary Budapest Hungary; ^10^ Zoology Unit The Finnish Museum of Natural History Helsinki Finland; ^11^ cE3C, Center for Ecology, Evolution and Environmental Change, Departamento de Biologia Animal Faculdade de Ciências, Universidade de Lisboa Lisboa Portugal; ^12^ Grimma Germany; ^13^ Area Avifauna Migratrice (BIO‐AVM), Istituto Superiore per la Protezione e la Ricerca Ambientale (ISPRA) Bologna Italy; ^14^ Center for Macroecology, Evolution and Climate, Natural History Museum od Denmark, University of Copenhagen Copenhagen Denmark

**Keywords:** annual variation, avian ecology, conservation, migratory birds, population abundance, productivity, survival rates

## Abstract

Synchronous fluctuations in species' abundance are influenced by synchrony in underlying rates of productivity and survival. However, it remains unclear how rate synchrony varies in space and time, contributes to abundance synchrony, and differs among species. Using long‐term annual count (number of adults captured), adult survival and productivity (number of juveniles captured per adult) data for breeding land‐birds at ringing sites across Europe, we show that synchrony is strongest and largest scale in productivity and weakest and smallest scale in counts. However, counts fluctuate more synchronously with survival than they do with productivity. These patterns hold for species which do not migrate or only migrate within Europe (European‐residents) and those migrating to sub‐Saharan Africa (subSaharan‐migrants), but the periodicity of productivity and survival synchrony is longer in European‐residents than in subSaharan‐migrants. This suggests that survival and productivity synchrony may interact to weaken abundance fluctuations but are influenced by environmental drivers operating over differing timescales in European‐resident and subSaharan‐migrant species.

## Graphical abstract

The strength and scale of synchrony in demographic rates of European bird populations is greatest in productivity, then survival, then counts. Counts fluctuate more synchronously with survival than with productivity and, despite having similar periodicities of count synchrony, productivity and survival synchrony generally occur over long timescales in European‐residents and short timescales in subSaharan‐migrants.

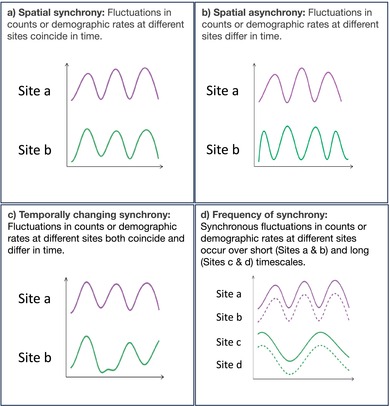

## 1 Introduction

Population synchrony, the correlated fluctuations in abundance among spatially discrete populations (Liebhold et al. 2004), is a central feature of the dynamics of wild populations and occurs in many taxa (Paradis et al. 2000; Liebhold et al. 2004; Defriez et al. 2016). Stronger synchrony is predicted to lead to greater population volatility or decline and to increased extinction risk due to reduced opportunities for demographic rescue (Heino et al. 1997; Palmqvist and Lundberg 1998). However, there is limited evidence of such effects in natural systems. The association between population synchrony and population decline is likely influenced by potentially complex effects of demographic synchrony on population synchrony (Schaub et al. 2015). However, while the contribution of demographic rates to population growth is well understood (Sæther and Bakke 2000; Robinson et al. 2014; Morrison, Robinson, Butler et al. 2016), the contribution of demographic synchrony to population synchrony remains largely unexplored (but see Schaub et al. 2015).

Synchrony in both population and demographic rates can be driven by multiple processes operating in isolation or in tandem and varying in space and time (Gouhier et al. 2010; Schaub et al. 2015). Correlated fluctuations in environmental conditions, termed Moran effects (Moran 1953), can drive both local correlations in population and/or demographic rates, and hence synchrony (Sæther et al. 2007), and also geographical variation in synchrony (Haynes et al. 2013). Equally, similarities in the timing and locations of seasonal movements by individuals could potentially lead to greater synchrony, for example through periods of favourable or unfavourable conditions being encountered by many individuals from across a population (Robinson et al. 2007; Pearce‐Higgins et al. 2015). Depending on the nature of the driver(s), synchrony may therefore vary in the distances over which it operates and occur over short and/or long periodicities within time‐series (Figure S1). However, whether a clear signal of these processes is present in population synchrony, and subsequent population trends, could depend on how synchrony in productivity and survival rates interacts in space and time; for example, if sites experience high and low productivity and survival during the same (in phase) or different (out of phase) years. Ultimately, population synchrony reflects species' sensitivities to environmental drivers (Morrison, Robinson and Pearce‐Higgins 2016; Herfindal et al. 2022), the spatial and temporal scales over which they operate and, critically, the demographic rate(s) through which they act; differences in synchrony between species are therefore likely to depend on their year‐round space use and the consequences for the environmental conditions they experience.

Opportunities to explore variation in and interactions between population and demographic synchrony over large spatial and temporal scales are rare as they require long time‐series of standardised survey data spanning numerous sites across large geographical areas. Across Europe, constant effort bird ringing sites (Euro‐CES) operate during the breeding season, and the resulting capture‐recapture data allow local counts, productivity, and survival rates to be quantified across a large suite of species (Robinson et al. 2009). We have previously shown (a) local co‐variation in site‐level population trends and in productivity, but not in survival (Morrison et al. 2021) and (b) that productivity varies more spatially than temporally, while survival shows more temporal than spatial variation (Morrison et al. 2022). This suggests contributions of both local fluctuations in productivity and larger‐scale fluctuations in survival to fluctuations in counts, but the extent of synchrony in demographic rates, the spatial and temporal scales over which they occur, and their relationships with population synchrony remain unclear.

Using data from Euro‐CES schemes for 26 European passerine breeding bird species, we estimate the scale (distance over which population fluctuations remain correlated) and strength (magnitude of the correlation at scale of zero; Jones et al. 2007) of synchrony in counts (number of individuals captured in each site in each year: used as the estimate of local population size), productivity and adult survival. In addition, we use wavelet analysis (Sheppard, Defriez, et al. 2019) to identify the periodicity of synchrony in counts and demographic rate(s) and how these vary in time and between species. We also use these approaches to explore the influence of demographic rate synchrony on count synchrony and the spatial scales over which this operates. Specifically, we determine how the (1) scale and strength, (2) congruence (within‐species synchrony between counts and demographic rates) and (3) periodicity of synchrony vary between counts, productivity and survival rates. We report our findings for all species and also separately for European‐resident and subSaharan‐migrant species to reflect the variation in exposure to potential environmental drivers associated with large‐scale differences in geographic distribution across the annual cycle between these two groups.

## 2 Materials and Methods

### 2.1 Demographic Data From the European Constant Effort Site Scheme (Euro‐CES)

Data were collated from 995 Euro‐CES sites, spanning 12 countries across Europe, all of which use standardised mist‐netting during the breeding season to measure the relative productivity and annual adult survival rates of passerine birds. At each Euro‐CES site, licensed ringers deploy a series of mist‐nets in the same positions, for the same length of time, during morning and/or evening visits, typically between April–May and July–August (the season starts and ends later at higher latitudes). Data submitted as part of the Euro‐CES scheme follow local guidelines for ageing individuals, typically on the basis of plumage characteristics, according to strict and standardised protocols and undertaken by experienced, qualified ringers (Svensson 2023). We only included years in which sites were (a) visited seven or more times in the season (including at least three visits in each of the first and second halves of the season), (b) had been running for five or more years and, only for estimates of productivity and apparent survival rates (hereafter survival) for each species, (c) on which two (the minimum needed to estimate these vital rates) or more adults had been captured in total, between 1998 and 2019. This therefore excludes sites that have either never caught an adult of that species or only caught one, as neither productivity nor survival rates could be estimated in these cases.

### 2.2 Classifying Migratory Status

Each species was classified as either ‘European‐resident’ (those that stay within Europe during the non‐breeding season) or ‘subSaharan‐migrant’ (species in which the majority of the European population covered by Pan‐European Common Bird Monitoring Scheme (PECBMS; https://pecbms.info) winters south of the Sahara; Table S2, see Vickery et al. 2014 for further details of classification).

### 2.3 Estimating Within‐Species Synchrony

To explore the impact of pooling data from survey locations over different spatial resolutions on estimates of synchrony, we estimated the scale and strength of synchrony for each species at resolutions of 25, 50 and 100 km^2^ (see Table S3 for details of all analytical procedures and equations).

### 2.4 Estimating Annual Abundance, Productivity and Survival Rates

All analyses were carried out in R version 4.2.1 (R Core Team 2022), and all model assumptions were checked by visually assessing residual plots; no assumptions were violated. For each species, annual estimates of count (number of adults captured), productivity (number of juveniles per adult) and adult apparent survival (capture‐recapture) were calculated within each 25, 50 and 100 km^2^ grid cell. As not all sites within each grid cell were surveyed in all years, we fitted General Linear Mixed Models (GLMMs), with site as a random effect (intercept only), except when there were five or fewer sites in a grid cell, in which case we fitted a General Linear Model (GLM) (Table S3).

To estimate annual variation in counts, we fitted models with a Poisson error structure and a log link, with the number of adult individuals caught per season as the response variable and the year fitted as a categorical fixed effect.

To estimate annual variation in productivity, we modelled the ratio of juvenile to adult birds caught in each season. Models were fitted with a binomial error structure, with the total number of juveniles as the response variable, the total number of birds (juveniles + adults) as the binomial denominator and year fitted as a categorical fixed effect.

To estimate adult apparent annual survival rates, we used the Cormack‐Jolly‐Seber (CJS) formulation of mark‐recapture models while accounting for transient individuals (residency probability; see Supporting Information—Data S1). We estimated: (a) *apparent survival probability*—the probability that a marked individual alive at sampling occasion t will survive and remain in the population (i.e., not permanently emigrate) between sampling occasion *t* and *t* + 1; and (b) *recapture probability*—the probability that a previously marked individual alive and associated with the population at time *t* will be captured. For each 25, 50 and 100 km^2^ grid cell, recapture probability was estimated as a fixed effect and year was the predictor variable fitted as a categorical fixed effect.

We fitted the survival models in a Bayesian framework, using (vague) priors for survival probabilities, recapture probabilities and residency probabilities (See Table S4). To summarise the posterior distribution of each parameter, we used the Markov‐chain Monte‐Carlo (MCMC) algorithm implemented in JAGS v.3.3.0, via the R package rjags (Plummer 2003). We computed two chains of 5000,000 iterations, of which we discarded the first 2,000,000 of each as ‘burn‐in’ and sampled every 5000th, resulting in a posterior sample of 1200 parameter estimates. We inspected the traceplots to ensure there was full coverage of the appropriate parameter space and convergence of the MCMC chains was assessed using the Gelman–Rubin statistic R‐hat (Brooks and Gelman 1998). Convergence was satisfactory for all parameters (R‐hat < 1.1).

### 2.5 Fitting the Correlograms

To estimate synchrony, we fitted spatial correlograms which provide a tool for spatially explicit exploration of synchrony and thus provide additional insights to those from range‐wide estimates for example, Interclass correlation coefficients (ICC) (Morrison et al. 2022; Ghislain et al. 2024). To remove temporal trends that can lead to spurious correlations while not removing potentially informative temporal autocorrelation (Buonaccorsi et al. 2001), we detrended the count and demographic data.

Detrending was carried out by fitting Gaussian GLMs, with the predicted annual estimates of count, productivity or survival for each grid cell as the response variable and year as a continuous explanatory variable. We extracted the residuals from these models to use as our detrended time‐series. Pearson's correlations of the estimated detrended time‐series were then carried out between all pairs of grid cells for which time‐series overlapped by 5 years or more. For each species, we used General Additive Models (GAMs), fitted in using the R package mgcv (Wood 2006), to estimate correlograms for each grid cell (25, 50 or 100 km^2^). The estimated correlation coefficients were modelled as a smoothed function of the distance between each pair of grid cells. GAMs were only fitted where there were 20 or more correlation coefficients. Model predictions and 95% confidence intervals were extracted using posterior simulation (Miller 2019) and used to estimate the scale and strength of synchrony. Scale was estimated as the distance at which the predicted value of the pair‐wise correlation coefficients equalled zero. The maximum distance a grid cell can be away from another grid cell depends on where it lies within the species' range. In order to control for differences in species' range shapes and sizes, we therefore calculated, for each grid cell, the maximum distance between this focal cell and all other grid cells and, for each species, identified the shortest of these maximum distances, hereafter termed minimum distance (where minimum distance represents half of the distance between the two most distant points where the species occurs within the survey area). For each species, at each resolution, any estimates of scale greater than this minimum distance were then replaced by this minimum distance. Strength was estimated as the predicted value of the pair‐wise correlation coefficients at zero distance. Maps of the resulting estimates of strength and relative scale of demographic and abundance synchrony for each species are available at https://synchrony.uea.ac.uk/.

At the species level, positive correlations between the estimated scale of synchrony and the minimum distance suggested a tendency for our estimates of scale to be restricted by range size (Figure S2). There was, however, a high degree of variation around this association, with high and low scales found across the range of minimum distance (Figure S2). To correct for any effect of range size, we therefore calculated an estimate of relative scale as the scale divided by the minimum distance and used this in our subsequent analyses. High and low relative scales also occurred across the full range of minimum distances (Figure S2).

### 2.6 Congruence of Synchrony

In addition to fitting correlograms to the count, productivity and survival estimates, we also fitted correlograms between count and productivity and between count and survival rate at each spatial resolution. When estimating the congruence of synchrony between count and productivity, count is lagged by a year, that is, count (of adults) in year *t* + 1 is related to productivity (ratio of juveniles to adults) in year *t* and when estimating the congruence of synchrony between count and survival, count in year *t* + 1 is related to adult survival between year *t* and *t* + 1. For each focal cell, Pearson's correlations of the estimated count detrended time‐series were carried out with the estimated survival or productivity detrended time‐series in all other grid cells. As above, these cell‐level correlation coefficients were then used as the response variable in GAMs and the relative scale and strength of synchrony between the rates (count‐survival and count‐productivity) extracted.

### 2.7 Periodicity of Synchrony

Wavelet analysis is used to detect periodic components in time‐series (Addison 2002). Here we use it to identify the periodicity of synchrony, that is, whether the frequency of synchrony has short or long timescales and how this varies through our time‐series (Figure S1d). All wavelet analyses were implemented in the open‐source ‘wsyn’ software package v.1.0.4 for the R language (Sheppard, Walter, et al. 2019). For each species, we characterised the synchrony of counts, productivity and survival rates using wavelet mean phasor field magnitudes (wpmf). We standardise our predicted annual estimates of count and rate in each grid cell using the *cleandat* function in ‘wsyn’, setting the clev option to 2 (time series are (individually) linearly detrended and de‐meaned). The resulting estimates were then used as input to the wpmf analysis. The output of the wpmf analysis is a series of plots which show the degree of synchrony among grid cells as a function of time and timescale (Figures S3–S5). The plots therefore show the strength of synchrony at each timescale for each year in the time series. We created composite wavelet plots for subSaharan‐migrant, European‐resident and all species by taking the average wavelet magnitude at each timescale and year combination for each group. The statistical significance of phase synchrony at a given time and timescale can be assessed through comparison to a null hypothesis of random phases (Sheppard et al. 2013, 2016; Sheppard, Walter, et al. 2019). We used a significance threshold of *p* < 0.05, which is indicated on the species‐level plots as contours (Figures S3–S5).

### 2.8 Variation in Synchrony Between Resolutions

To explore the impact of spatial resolution on estimates of synchrony, we fitted GLMMs with species‐level estimates of relative scale or strength of synchrony as the response variables and resolution (25, 50, 100 km^2^) as the explanatory variable. Grid cell and species were fitted as random effects to account for variation in species distributions across sites. To account for precision error in our estimates of synchrony, we included weights in all models. Models where relative scale was fitted as the response variable were weighted by the inverse of the mean of the standard error around the values predicted by the GAM between 0 distance and the scale. Models where strength was the response variable were weighted by the inverse of the size of the confidence interval around the estimates of strength. As differences in the scale and strength of synchrony between resolutions were relatively minor (Figure S6 and Table S5), only the 100 km^2^ estimates were used in subsequent analyses.

### 2.9 Variation in the Relative Scale and Strength of Synchrony

To explore variation in synchrony between demographic rates and whether these varied between European‐resident and subSaharan‐migrant species, we fitted separate GLMMs with the species‐level weighted estimates of relative scale or strength as response variables, demographic rate and migratory status as fixed effects and grid cell and species as random effects.

### 2.10 Variation in the Congruence of Synchrony

To explore variation in the congruence of count‐rate synchrony, we fitted GLMMs with species‐level weighted estimates of relative scale and strength as response variables, congruence pairing (count‐survival or count‐productivity), migratory status and their interaction as explanatory variable effects, and grid cell and species as random effects.

### 2.11 Variation in the Periodicity of Synchrony

To explore variation in the timescale of periodicity in the demographic metrics, for each species we fitted binomial GLMs, with the number of years in which the wavelet phasor mean field magnitudes were statistically significant in each timescale modelled with the total number of years as the binomial denominator. Timescale, demographic metric, migratory status, and their two‐way interactions were fitted as response variables. We used a quasibinomial error structure to account for overdispersion.

## 3 Results

### 3.1 Variation in the Scale and Strength of Synchrony

**FIGURE 1:**
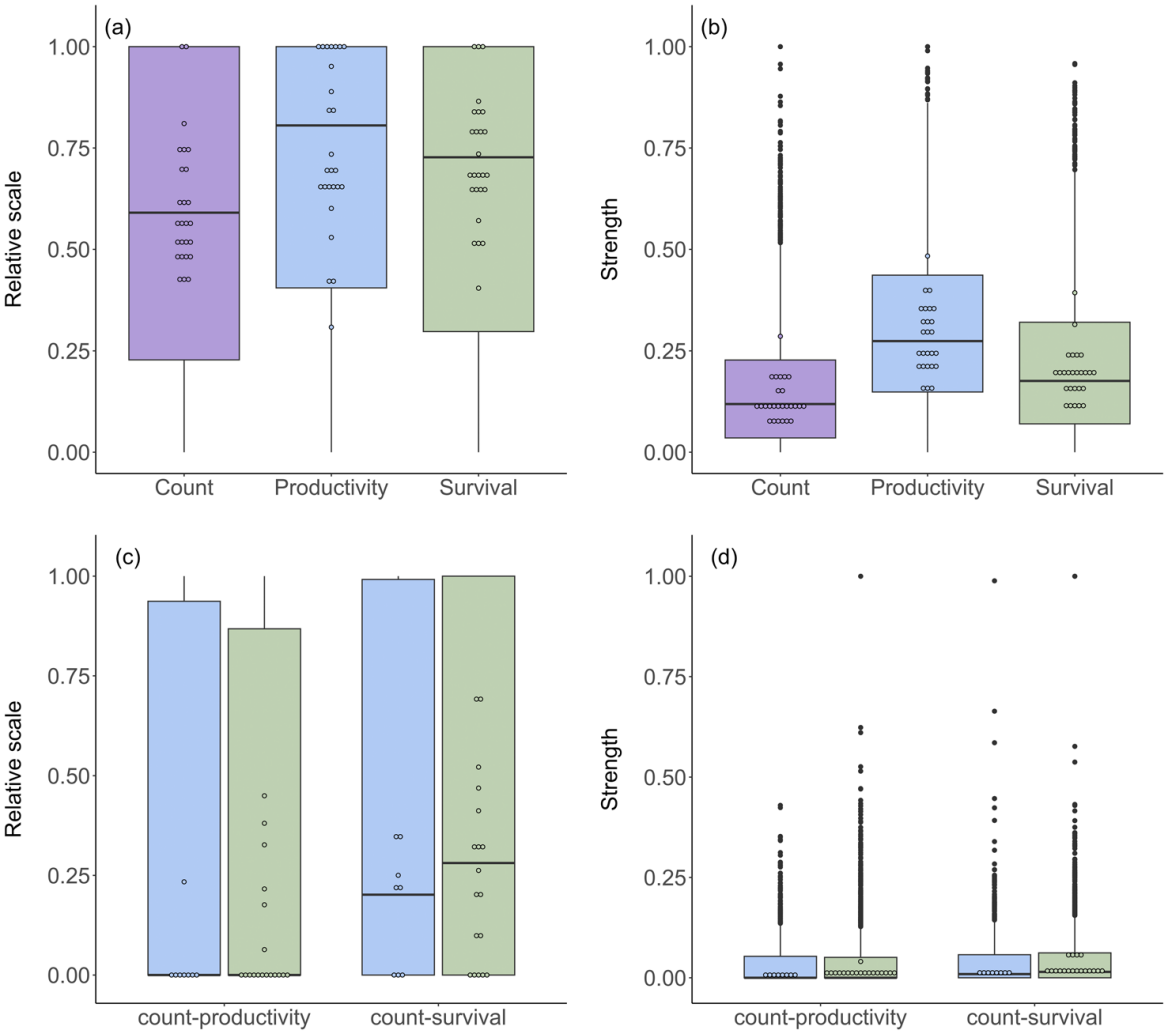
Variation in estimates of relative scale (a, c) and strength (b, d) of synchrony in count, productivity and adult survival rates across 26 species (a, b), and in count‐productivity and count‐survival congruence (c, d) of synchrony in subSaharan‐migrant (blue) and European‐resident (green) species, across Europe between 1998 and 2019. Circles show the median species‐level estimates. Horizontal bars indicate medians, boxes indicate interquartile range (IR), lower whisker indicates 1st quartile (−1.5×IR), upper whisker indicates 3rd quartile (+1.5×IR), black dots indicate outliers (species‐grid square values 1.5 times higher or lower than 1st and 3rd quartile respectively). See Figure S7 for histograms of underlying data. SubSaharan‐migrant species are those primarily wintering south of the Sahara and European‐resident species are those that primarily stay in Europe year‐round.

On average, synchrony in productivity of European land‐birds occurs across larger relative scales and is stronger than in survival rates (productivity mean relative scale = 0.66 (CIs: 0.64–0.70), survival = 0.61 (0.58–0.64); productivity mean strength = 0.32 (0.30–0.33), survival = 0.23 (0.22–0.25), Figure 1a,b, Table 1 and Tables S6–S8). Count synchrony was weaker and occurred over smaller relative scales than both demographic rates (count mean relative scale = 0.57 (0.54–0.60), mean strength = 0.16 (0.14–0.18), Figure 1a,b, Table 1 and Table S6, Figure S7). Neither the relative scale nor strength of synchrony in count and demographic rates differed significantly between subSaharan‐migrant and European‐resident species (Table 1).

**TABLE 1:** Results of GLMMs of variation in relative scale and strength of synchrony in counts, productivity and survival rates in subSaharan‐migrant and European‐resident species across Europe between 1998 and 2019.

Synchrony metric (response)	Explanatory	*X* ^2^	df	Pr(> *X* ^2^)
Relative scale	Count/rate metric	92.94	2	**< 0.001**
	Migratory status	0.006	1	0.94
Strength	Count/rate metric	1048.6	2	**< 0.001**
	Migratory status	0.156	1	0.69

*Note:* subSaharan‐migrant species are those primarily wintering south of the Sahara and European‐resident species are those that primarily stay in Europe year‐round. The numbers in bold are significant at the 0.05 level.

### 3.2 Variation in the Congruence of Synchrony

Even though average synchrony in survival rates is at a smaller scale and weaker than in productivity (Figure 1a,b), the association between annual fluctuations in count and survival occurs across significantly larger relative spatial scales than associations between annual fluctuations in count and productivity (Figure 1c, Table 2) and is (weakly) significantly stronger (Figure 1d, Table 2). Furthermore, synchrony between count and survival rates occurred across significantly larger relative scales in European‐resident (median = 28% of range size) than subSaharan‐migrant species (median = 20% of range size) but was not stronger (Figure 1c, Table 2).

**TABLE 2:** Results of GLMMs of variation in relative scale and strength of synchrony in congruence between counts and rates (count—productivity vs. count—survival) in subSaharan‐migrant and European‐resident species across Europe between 1998 and 2019.

Synchrony metric (response)	Explanatory	*X* ^2^	df	Pr(> *X* ^2^)
Relative scale	Congruence pairing	34.57	1	**< 0.001**
	Migratory status	0.08	1	0.78
	Congruence pairing × Migratory status	4.98	1	**0.03**
Strength	Congruence pairing	4.30	1	**0.04**
	Migratory status	1.07	1	0.30
	Congruence pairing × Migratory status	0.01	1	0.91

*Note:* subSaharan‐migrant species are those primarily wintering south of the Sahara, and European‐resident species are those that primarily stay in Europe year‐round. The numbers in bold are significant at the 0.05 level.

### 3.3 Variation in the Periodicity of Synchrony

**FIGURE 2:**
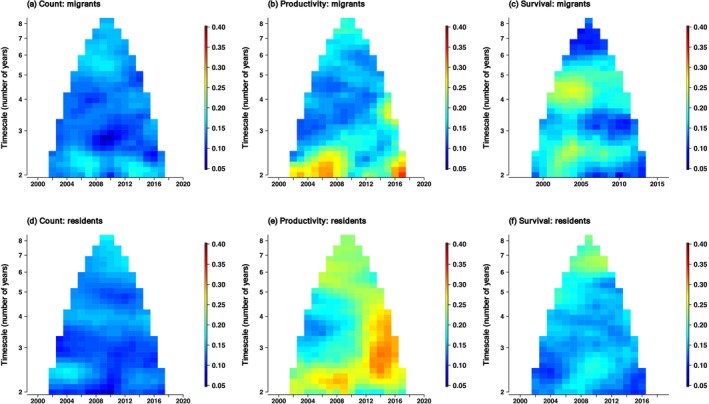
Composite wavelet phasor mean field magnitudes of count (a, d), productivity (b, e) and survival rates (c, f) for subSaharan‐migrant (a–c) and European‐resident species (d–f) across Europe between 1998 and 2019. Higher values indicate greater synchrony at the indicated times (year) (*x*‐axis) and periodicities (number of years) (*y*‐axis); for individual species, see Figures S3–S5. SubSaharan‐migrant species are those primarily wintering south of the Sahara and European‐resident species are those that primarily stay in Europe year‐round.

**FIGURE 3:**
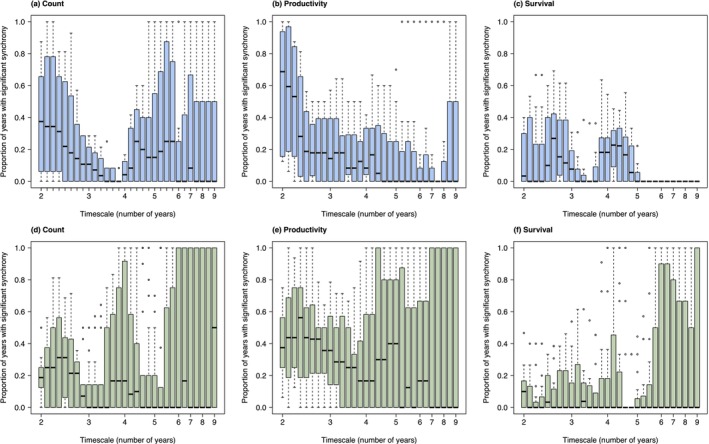
Species‐level variation in the proportion of years with significant synchrony that operates for different timescales (periodicities), in counts (a, d), productivity (b, e) and survival rates (c, f), in subSaharan‐migrant (a–c, blue, *n* = 8) and European‐resident species (d–f, green, *n* = 18) across Europe between 1998 and 2019. SubSaharan‐migrant species are those primarily wintering south of the Sahara and European‐resident species are those that primarily stay in Europe year‐round.

Overall, wavelet analysis confirmed the correlogram finding that synchrony was strongest in productivity, intermediate in survival and weakest in counts (Figures 2 and 3, Table 3, Table S9). In subSaharan‐migrant species, short‐timescale (2–3 years) synchrony occurred in counts, productivity and survival rates, although it was strongest in productivity (Figures 2 and 3, Table 3). Long‐timescale (> 6 years) synchrony was also present in counts and productivity of subSaharan‐migrant species, but not in survival (Figures 2 and 3). By contrast, in European‐resident species, synchrony in counts and productivity was most frequent at short (2–3 year) timescales, but also occurred at intermediate (3–5 years) and long (> 6 years) timescales. Synchrony in survival of European‐resident species occurred most commonly at long (> 6 years) timescales (Figures 2 and 3).

**TABLE 3:** Results of a binomial GLM of variation in the proportion of years with significant synchrony at different timescales in counts, productivity, and survival rates in subSaharan‐migrant and European‐resident species across Europe between 1998 and 2019.

	*X* ^2^	df	*p*
Timescale	0.002	1	0.96
Count/rate metric	201.94	2	**< 0.001**
Migratory status	10.26	1	**< 0.001**
Timescale × count/rate metric	26.97	2	**< 0.001**
Timescale × migratory status	24.57	1	**< 0.001**
Count/rate metric × migratory status	16.52	2	**< 0.001**

*Note:* subSaharan‐migrant species are those primarily wintering south of the Sahara, and European‐resident species are those that primarily stay in Europe year‐round. The numbers in bold are significant at the 0.05 level.

## 4 Discussion

Citizen science data for 26 species of European‐resident and subSaharan‐migratory European breeding passerine birds surveyed over a two‐decade period (1998–2019) reveal that synchrony in productivity operates over greater spatial scales and is stronger, on average, than synchrony in adult survival rates. However, synchrony in counts was consistently lower than in both productivity and survival, suggesting that productivity and abundance synchrony interact to weaken synchrony in counts. Congruence of synchrony between counts and survival rates was larger‐scale (especially for European‐resident species) and stronger than congruence of synchrony between counts and productivity. Synchrony in both demographic rates occurred more commonly over long timescales in European‐resident species and short timescales in subSaharan‐migrant species. This suggests a greater contribution to count synchrony of (weaker, smaller‐scale) synchrony in survival rates than (stronger, larger‐scale) synchrony in productivity in both European‐resident and subSaharan‐migrant species, despite their differing periodicities of rate synchrony.

The relatively strong, large‐scale (approaching half of the breeding range, on average) synchrony in productivity suggests that, during the breeding season, key environmental conditions are correlated over a large proportion of species' European ranges. This could be underpinned by processes such as consistent weather conditions occurring over large areas, and/or local drivers (e.g., agricultural practices) operating in a regionally consistent way (Morrison et al. 2010), and influencing for example, clutch sizes or survival rates of eggs, nestlings and/or juveniles during the post‐fledgling period. For adult survival rates, synchrony occurred over significantly smaller scales than synchrony in productivity. However, it should be noted that annual estimates of survival rates have greater uncertainty than annual estimates of productivity. Propagating this error into our synchrony models is not currently possible, but could potentially improve estimates of the effects of survival synchrony on count synchrony. National‐level synchrony in survival rates of European landbirds has previously been reported (Ghislain et al. 2024) and has been linked to extreme and large‐scale weather events. In addition, the large‐scale droughts in the Sahel region of Africa during the 1970s were associated with dramatic declines in the annual survival of Sedge Warblers *Acrocephalus schoenobaenus* across the UK (Peach et al. 1991), while cold winters have been strongly associated with widespread (short‐term) reductions in the numbers of Wrens *Troglodytes troglodytes* (Robinson et al. 2007; Morrison, Robinson, Pearce‐Higgins et al. 2016) and lower survival rates of juvenile little owls *Athene noctua* (Perrig et al. 2024). However, as survival rates can be influenced by the environmental conditions encountered throughout the annual cycle, the potential counteracting effects of conditions in differing parts of the cycle could also be expected to weaken overall survival synchrony. Indeed, our finding of relatively small‐scale and weak synchrony in survival rates suggests that, in our time series at least, the frequency of extreme weather events and the number of species impacted are generally low. Further exploration of the strength of abundance, productivity and survival synchrony at different relative distances (i.e., from distance = 0 to distance = scale) could potentially help identify important environmental drivers. For example, a rapid decline in synchrony strength with distance would suggest a role for local‐scale (e.g., land‐use) drivers alone, whilst evidence of a bimodal pattern, with peaks in synchrony strength at both small and large scales, might suggest synchrony is being driven by both local and large‐scale (e.g., weather patterns) factors.

Population growth rates of short‐lived passerine birds such as the species included in this study are typically more sensitive to variation in survival rates than productivity (Robinson et al. 2014; Sæther et al. 2016), and this may explain the greater congruence of synchrony between counts and survival than between counts and productivity. Simulation modelling has shown that synchrony in rates of immigration and apparent survival is able to induce population synchrony, but synchrony in local productivity does not (Schaub et al. 2015). It should be noted that, in passerines at least, the impact of productivity synchrony on abundance synchrony may be limited by both post‐fledging mortality and movement, including sex‐specific dispersal, as rates of natal dispersal are typically higher in females than males (Pusey 1987). In addition, analyses of sex biases in willow warbler, *Phylloscopus trochilus* , populations in relation to local abundance suggest that females preferentially recruit into larger populations (Morrison, Robinson, Clark et al. 2016). The lack of evidence of strong congruence of synchrony between counts and productivity may reflect the complexity of the indirect mechanisms operating and interacting to influence variation in species abundance. The weaker congruence of synchrony between count and survival rates in subSaharan‐migrants than European‐residents could also reflect the weak migratory connectivity that is typical of migratory systems (Finch et al. 2017), as individuals that migrate between Europe and sub‐Saharan Africa are more likely to experience different conditions throughout the year. Exploration of the pattern of change in synchrony strength with distance, both for abundance, survival, and productivity individually (as discussed above) and for their congruence, might also indicate whether the relative contribution of productivity and survival synchrony to population synchrony changes with spatial scale.

Despite some European‐resident species migrating within Europe, we found differing periodicities of demographic synchrony in subSaharan‐migratory and European‐resident species. This suggests differing roles of environmental processes in driving synchrony in these species, which are likely to be caused by conditions outside of Europe rather than migration per se and may provide new insights into the evolution of migratory strategies (Somveille et al. 2015). Annual mixing of migratory individuals from across populations may reduce the impact of environmental processes operating over long timescales, whereas for European‐resident species, individuals from the same populations may experience more similar annual conditions and hence respond to environmental processes operating over both short and long timescales. More pressingly, if environmental changes decouple the links between productivity and survival synchrony that currently dampen count synchrony, this may lead to an increase in the count fluctuations predicted to increase extinction risk (Heino et al. 1997). Indeed, it is possible that this may be a contributory factor to the widespread decline of subSaharan‐migrant species (Vickery et al. 2014) but this requires further exploration.

## 5 Conclusions

Synchrony in counts, productivity and adult survival is evident in the population dynamics of landbirds across Europe, with interactions between the demographic rates potentially dampening count synchrony. The differing periodicities of synchrony in subSaharan‐migrant and European‐resident species also suggest that the population dynamics of subSaharan‐migrant and European‐resident species are sensitive to different drivers, which may influence how these species respond to changing environmental conditions. Identifying the processes driving synchrony in each rate will be a key step in developing an understanding of their direct and interactive contributions to count synchrony and population trends, and to identifying actions capable of recovering the many species currently in decline across Europe.

## Author Contributions

**Catriona A. Morrison:** conceptualisation, formal analysis, methodology, project administration, writing – original draft and writing – review and editing; **Simon J. Butler:** conceptualisation, funding acquisition, project administration, methodology, supervision, writing – original draft and writing – review and editing; **Jennifer A. Gill:** conceptualisation, funding acquisition, methodology, supervision, writing – original draft and writing – review and editing; **Claire Buchan:** data management and handling. **Robert A. Robinson:** conceptualisation, formal analysis, methodology. **Juan Arizaga:** data curation; **Oriol Baltà:** data curation; **Emanuel Baltag:** data curation; **Jaroslav Cepák:** data curation; **Pierre‐Yves Henry:** data curation; **Ian Henshaw:** data curation; **Zsolt Karcza:** data curation; **Petteri Lehikoinen:** data curation; **Ricardo Jorge Lopes:** data curation; **Bert Meister:** data curation; **Simone Pirrello:** data curation; **Kasper Thorup:** data curation.

### Peer Review

The peer review history for this article is available at https://www.webofscience.com/api/gateway/wos/peer‐review/10.1111/ele.70105.

## Supporting information


Data S1.

## Data Availability

The R code and example supporting datasets for this manuscript are available on dryad: DOI https://doi.org/10.5061/dryad.pnvx0k709 and github https://github.com/catmorrison/Synchrony.
