# Supplementary material for: How Do Synchrony in Survival and Productivity Influence Abundance Synchrony in European Landbirds?
Source: Ecol Lett. 2025 May 13;28(5):e70105. doi: 10.1111/ele.70105 (PMC12070856; doi:10.1111/ele.70105)
Supplement: Supplementary file 1 — Data S1. [file ELE-28-0-s001.docx]

**Figure S1**: Fluctuations in abundance or demographic rates at different sites can occur a) synchronously, b) asynchronously, c) synchronously and asynchronously in time and d) synchronously over short and long periodicities.

**
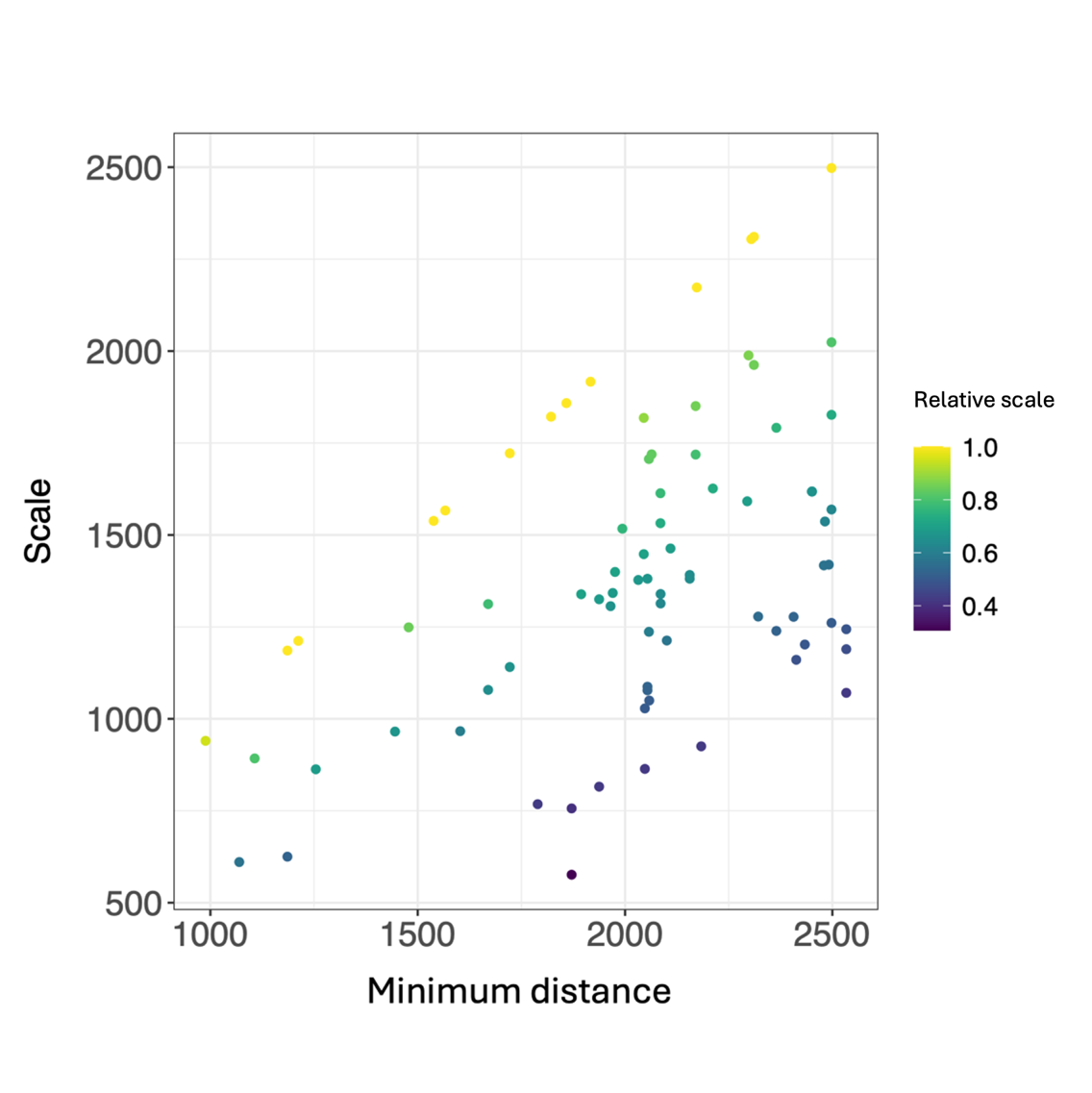
**

**Figure S2:** The association between scale (r = 0.47, p <0.001) and the minimum distance. Colours indicate the relative scale (scale/minimum distance).

**Figure S3:** Species-level wavelet phasor mean field magnitudes of counts. Higher values indicate greater synchrony at the indicated times and timescales. Contours indicate statistical significance of phase synchrony at p = 0.05. Note the scale of the z-axis varies between species.

**Figure S4:** Species-level wavelet phasor mean field magnitudes of productivity. Higher values indicate greater synchrony at the indicated times and timescales. Contours indicate statistical significance of phase synchrony at p = 0.05. Note the scale of the z-axis varies between species.

**Figure S5:** Species-level wavelet phasor mean field magnitudes of survival rates. Higher values indicate greater synchrony at the indicated times and timescales. Contours indicate statistical significant of phase synchrony at p = 0.05. Note the scale of the z-axis varies between species.

**Figure S6:** Variation in estimates of scale (a,b), relative scale (c,d) and strength (e,f) in count, productivity and survival rates across three spatial resolutions of analysis (100km^2^, 50km^2^, 25km^2^).

**Figure S7:** Frequency distributions of estimates of relative scale (a-c) and strength (d-f) in count (a&d), productivity (b&e) and survival (c&f) of 26 species across 200 100km^2^ grid cells.

**Table S1:** The number of sites, seasonal visits, species, in each of the European Constant Effort Site Schemes from 1998 - 2019 that contributed to count and demographic models.

| **Scheme** | **Number of sites** | **Number of visits** | **Number of species** | **Range of time series** |
| --- | --- | --- | --- | --- |
| Britain & Ireland | 207 | 12 | 26 | 1998 - 2019 |
| Czech Republic | 39 | 9 | 25 | 2004 – 2019 |
| Denmark | 7 | 12 | 24 | 2004 - 2019 |
| Finland | 55 | 12 | 24 | 2013 - 2019 |
| France | 9 | 9 | 25 | 1998 - 2019 |
| Germany | 84 | 12 | 25 | 2000 - 2019 |
| Hungary | 39 | 9 | 24 | 2004 - 2019 |
| Italy | 21 | 12 | 20 | 2015 - 2019 |
| Netherlands | 68 | 12 | 26 | 1994 - 2019 |
| Portugal | 6 | 12 | 20 | 2007 - 2019 |
| Spain (San Sebastian) | 13 | 7 | 23 | 2010 - 2019 |
| Spain (Madrid) | 73 | 12 | 23 | 1999 - 2019 |
| Spain (Catalonia) | 69 | 10 | 22 | 1991- 2019 |
| Sweden | 33 | 12 | 23 | 2004 - 2019 |

**Table S2:** Migratory status of species used in analysis (extracted from Vickery *et al.* 2014 and Wilman *et al.* 2016; European-resident = stays in Europe, subSaharan-migrant = leaves Europe) and the number of Euro-CES sites in which species were found in sufficient numbers to include in the analysis.

| **Latin name** | **English name** | **Euring code** | **Migratory status** | **Number of Euro-CE sites** |
| --- | --- | --- | --- | --- |
| *Acrocephalus palustris* | Marsh warbler | 12500 | subSaharan-migrant | 438 |
| *Acrocephalus schoenobaenus* | Sedge warbler | 12430 | subSaharan-migrant | 852 |
| *Acrocephalus scirpaceus* | Reed warbler | 12510 | subSaharan-migrant | 938 |
| *Aegithalos caudatus* | Long-tailed tit | 14370 | European-resident | 921 |
| *Carduelis carduelis* | Goldfinch | 16530 | European-resident | 892 |
| *Carduelis chloris* | Greenfinch | 16490 | European-resident | 948 |
| *Cettia cetti* | Cettis warbler | 12200 | European-resident | 794 |
| *Emberiza citrinella* | Yellowhammer | 18570 | European-resident | 542 |
| *Emberiza schoeniclus* | Reed bunting | 18770 | European-resident | 700 |
| *Erithacus rubecula* | Robin | 10990 | European-resident | 945 |
| *Fringilla coelebs* | Chaffinch | 16360 | European-resident | 930 |
| *Luscinia megarhynchos* | Common nightingale | 11040 | subSaharan-migrant | 753 |
| *Parus caeruleus* | Blue tit | 14620 | European-resident | 941 |
| *Parus major* | Great tit | 14640 | European-resident | 950 |
| *Passer domesticus* | House sparrow | 15910 | European-resident | 786 |
| *Phylloscopus collybita* | Chiffchaff | 13110 | European-resident | 929 |
| *Phylloscopus trochilus* | Willow warbler | 13120 | subSaharan-migrant | 918 |
| *Prunella modularis* | Dunnock | 10840 | European-resident | 790 |
| *Pyrrhula pyrrhula* | Bullfinch | 17100 | European-resident | 650 |
| *Sylvia atricapilla* | Blackcap | 12770 | European-resident | 981 |
| *Sylvia borin* | Garden warbler | 12760 | subSaharan-migrant | 953 |
| *Sylvia communis* | Whitethroat | 12750 | subSaharan-migrant | 925 |
| *Troglodytes troglodytes* | Wren | 10660 | European-resident | 866 |
| *Turdus merula* | Blackbird | 11870 | European-resident | 991 |
| *Turdus philomelos* | Song thrush | 12000 | European-resident | 895 |

**Table S3:** Descriptions of all analytical steps, associated methods and equations used in the analyses of synchrony in count, survival and productivity at different spatial resolutions

| **Analysis step** | **Method** |
| --- | --- |
| 1) For each 100 km^2^, 50 km^2^ and 25 km^2^ grid cell, calculate annual mean estimates of:   - Count (number of adult birds captured), - Survival (adult capture-recaptures) - Productivity (number of juveniles per adult) | For all grid cells with > 5 sites, fit a GLMM (Eq 1); for all cells with 5 or fewer sites, fit a GLM (Eq 2):  Eq 1. **GLMM**: g(*θ_i_*) = ⍺*_j[i]_* + β_1_ × year*_i_* (categorical) + ԑ*_i_*  Eq 2. **GLM:** g(*θ_i_*) = ⍺ + β_1_ × year*_i_* (categorical) + ԑ*_i_*  *θ =* Count or rate.  j = site  ⍺*_j_*~ N(µ_α_,σ^2^_α_)  ԑ*_i_*~ N(0, σ^2^)  Count: g() = log link and Poisson distribution  Productivity and survival: g() = logit link and binomial distribution |
| 2) Detrend annual estimates of count, survival and productivity | a) Fit a GLM (Eq3) to the annual estimates produced in step 1:  Eq 3. **GLM**: g(*θ_i_*) = ⍺ + β_1_ × year*_i_* (continuous) + ԑ*_i_*  ԑ*_i_*~ N(0, σ^2^)  g() = identity link and Gaussian distribution  b) Extract the residuals from these models to use in detrended time-series |
| 3) Calculate Pearson’s correlations between detrended time-series | For all pairs of grid cells for which time-series overlapped by five years or more, calculate Pearson’s correlations of the detrended time-series |
| 4) Fit spatial correlograms | For each grid cell with 20 or more correlations, fit a GAM (Eq 4):  Eq 4. **GAM:** g(*θ_i_*) = ⍺ + s(distance) + ԑ*_i_*  *θ =* Pearson’s correlations  S() = smoothed function  ԑ*_i_*~ N(0, σ^2^) |
| 5) Estimate strength and scale of synchrony for each grid cell | Using the predicted values from the GAM, extract the strength and scale of synchrony:  **Strength** = predicted value of the pair-wise correlation coefficients at distance = zero  **Scale** = the distance at which the predicted value of the pair-wise correlation coefficients equals zero |
| 6) Calculate maximum distance and relative scale | **Maximum distance =** for each grid cell, calculate the maximum distance to all other cells (with 20 or more correlations) and find the shortest distance value  **Relative scale =** Scale/(shortest)Maximum distance |

**Table S4:** The prior distributions used in the estimation of adult survival rates and productivity. tau.survival and tau.recapture are the precision of the site-level survival and recapture rates respectively and calculated as: 1/Variance in site-level rate^2^.

| **Fixed effects** | **Prior distribution** |
| --- | --- |
| Annual survival probability | dbeta(1,1) |
| Recapture probability | dbeta(1,1) |
| Slope of seasonal recapture probability | dnorm(0, 0.001) |
| Residency probability | dbeta(1,1) |
| **Random effects** |  |
| Site-level survival rate | dnorm(0,tau.survival) |
| Variance in site-level survival rate | dunif(0.0001,10) |
| Site-level recapture probability | dnorm(0,tau.recapture) |
| Variance in site-level recapture probability | dunif(0.0001,10) |

**Table S5:** Results of GLMMs of variation in relative scale and strength in counts, productivity, and survival rates across three spatial resolutions of analysis (25, 50, 100 km^2^).

| **Demographic metric** | **Response** | **Explanatory** | **Chisq** | **Df** | **Pr(>Chisq)** |
| --- | --- | --- | --- | --- | --- |
| Count | Relative scale | Resolution | 5.77 | 1 | 0.16 |
|  | Strength | Resolution | 226.8 | 1 | <0.001 |
| Productivity | Relative scale | Resolution | 0.40 | 1 | 0.53 |
|  | Strength | Resolution | 115.38 | 1 | <0.001 |
| Survival | Relative scale | Resolution | 4.59 | 1 | 0.03 |
|  | Strength | Resolution | 104.27 | 1 | <0.001 |

**Table S6** Post-hoc comparisons of differences in synchrony metrics between counts and demographic rates for 26 landbird species across Europe between 1998 and 2019.

| Synchrony metric | Difference | Estimate | SE | df | z-ratio | p-value |
| --- | --- | --- | --- | --- | --- | --- |
| Relative scale | Count – productivity | -0.10 | 0.10 | 7634 | 9.63 | **<0.001** |
|  | Count – survival | -0.04 | 0.01 | 7628 | 4.047 | **<0.001** |
|  | Productivity – survival | 0.06 | 0.01 | 7429 | -4.85 | **<0.001** |
| Strength | Count – productivity | -0.16 | 0.005 | 7612 | 32.25 | **<0.001** |
|  | Count – survival | -0.07 | 0.005 | 7596 | 14.61 | **<0.001** |
|  | Productivity – survival | 0.09 | 0.006 | 7434 | -15.35 | **<0.001** |

**Table S7:** Results of species-level GLMMs of differences in relative scale of synchrony between counts and productivity, counts and survival rates and survival rates and productivity (significant differences highlighted in bold).

| **Relative scale** | | | | | | | | |
| --- | --- | --- | --- | --- | --- | --- | --- | --- |
| **Count - productivity** | | | **Count - survival** | | | **Survival - productivity** | | |
| **Species** | **Count - productivity** | **p-value** | **Species** | **Count - survival** | **p-value** | **Species** | **Survival - productivity** | **p-value** |
| Blue tit | **-0.25** | **<0.001** | Common nightingale | **-0.19** | **<0.001** | Sedge warbler | **-0.32** | **<0.001** |
| Cettis warbler | **-0.23** | **<0.001** | Chiffchaff | **-0.18** | **<0.001** | Blackbird | **-0.26** | **<0.001** |
| Sedge warbler | **-0.22** | **<0.001** | Whitethroat | **-0.18** | **0.04** | Reed warbler | **-0.24** | **<0.001** |
| Common nightingale | **-0.21** | **<0.001** | Reed bunting | **-0.12** | **0.02** | Cettis warbler | **-0.18** | **<0.001** |
| Reed warbler | **-0.20** | **<0.001** | Robin | -0.12 | 0.06 | Yellowhammer | -0.17 | 0.08 |
| Blackbird | **-0.20** | **<0.001** | Great tit | **-0.10** | **0.03** | Blue tit | **-0.17** | **<0.001** |
| Yellowhammer | **-0.16** | **0.02** | Blackcap | **-0.10** | **0.03** | Bullfinch | **-0.12** | **0.01** |
| Blackcap | **-0.15** | **<0.001** | Blue tit | -0.08 | 0.07 | Lesser whitethroat | -0.09 | 0.07 |
| Reed bunting | **-0.15** | **0.01** | Dunnock | -0.08 | 0.11 | Willow warbler | -0.07 | 0.31 |
| Willow warbler | -0.13 | 0.06 | Long-tailed tit | -0.07 | 0.15 | Blackcap | -0.07 | 0.09 |
| Long-tailed tit | **-0.11** | **0.02** | Cettis warbler | -0.06 | 0.16 | Garden warbler | -0.05 | 0.32 |
| Whitethroat | -0.10 | 0.20 | House sparrow | -0.06 | 0.29 | Chaffinch | -0.04 | 0.38 |
| Great tit | **-0.10** | **0.03** | Greenfinch | -0.05 | 0.33 | Long-tailed tit | -0.04 | 0.40 |
| Lesser whitethroat | -0.09 | 0.05 | Willow warbler | -0.05 | 0.44 | Wren | -0.03 | 0.77 |
| Chiffchaff | -0.07 | 0.12 | Garden warbler | -0.02 | 0.71 | Reed bunting | -0.01 | 0.79 |
| Chaffinch | -0.06 | 0.18 | Yellowhammer | 0.00 | 0.98 | Common nightingale | -0.01 | 0.85 |
| Garden warbler | -0.06 | 0.19 | Chaffinch | 0.00 | 0.93 | House sparrow | 0.00 | 0.94 |
| Dunnock | -0.06 | 0.22 | Lesser whitethroat | 0.01 | 0.86 | Great tit | 0.00 | 0.96 |
| Robin | -0.05 | 0.42 | Marsh warbler | 0.03 | 0.70 | Dunnock | 0.01 | 0.88 |
| House sparrow | -0.05 | 0.27 | Reed warbler | 0.03 | 0.59 | Greenfinch | 0.02 | 0.78 |
| Bullfinch | -0.04 | 0.29 | Blackbird | 0.04 | 0.30 | Marsh warbler | 0.04 | 0.59 |
| Greenfinch | -0.03 | 0.53 | Goldfinch | 0.06 | 0.16 | Song thrush | 0.04 | 0.66 |
| Marsh warbler | 0.08 | 0.23 | Song thrush | 0.07 | 0.46 | Whitethroat | 0.06 | 0.44 |
| Song thrush | 0.10 | 0.23 | Bullfinch | 0.07 | 0.14 | Robin | 0.06 | 0.33 |
| Goldfinch | 0.10 | 0.05 | Sedge warbler | 0.09 | 0.20 | Goldfinch | 0.08 | 0.08 |
| Wren | **0.16** | **0.03** | Wren | **0.21** | **0.02** | Chiffchaff | 0.10 | 0.05 |

**Table S8:** Results of species-level GLMMs of differences in strength of synchrony between counts and productivity, counts and survival rates and survival rates and productivity (significant differences highlighted in bold).

| **Strength** | | | | | | | | |
| --- | --- | --- | --- | --- | --- | --- | --- | --- |
| **Count - productivity** | | | **Count - survival** | | | **Survival - productivity** | | |
| **Species** | **Count - productivity** | **p-value** | **Species** | **Count - survival** | **p-value** | **Species** | **Survival - productivity** | **p-value** |
| Song thrush | **-0.29** | **<0.001** | Chiffchaff | **-0.19** | **<0.001** | Sedge warbler | **-0.24** | **<0.001** |
| Marsh warbler | **-0.27** | **<0.001** | Common nightingale | **-0.19** | **<0.001** | Blackbird | **-0.23** | **<0.001** |
| Cettis warbler | **-0.26** | **<0.001** | Whitethroat | **-0.16** | **0.03** | Yellowhammer | -0.20 | 0.05 |
| Sedge warbler | **-0.25** | **<0.001** | Robin | **-0.15** | **0.03** | Reed warbler | **-0.19** | **<0.001** |
| Chiffchaff | **-0.21** | **<0.001** | Reed bunting | **-0.12** | **0.02** | Cettis warbler | **-0.17** | **<0.001** |
| Reed warbler | **-0.21** | **<0.001** | Greenfinch | **-0.10** | **0.04** | Blue tit | **-0.14** | **<0.001** |
| Wren | **-0.19** | **<0.001** | Blackcap | **-0.10** | **0.03** | Bullfinch | **-0.13** | **0.01** |
| Blackbird | **-0.19** | **<0.001** | Blue tit | -0.09 | 0.05 | Lesser whitethroat | -0.10 | 0.06 |
| Common nightingale | **-0.17** | **<0.001** | Dunnock | -0.09 | 0.07 | Willow | -0.09 | 0.22 |
| Lesser whitethroat | **-0.17** | **<0.001** | Cettis warbler | -0.09 | 0.06 | Blackcap | -0.05 | 0.24 |
| Goldfinch | **-0.17** | **<0.001** | Great tit | -0.08 | 0.09 | Chaffinch | -0.04 | 0.38 |
| Reed bunting | **-0.17** | **<0.001** | Long-tailed tit | -0.08 | 0.13 | Long-tailed tit | -0.04 | 0.43 |
| Willow warbler | **-0.17** | **<0.001** | House sparrow | -0.06 | 0.23 | Garden warbler | -0.03 | 0.51 |
| Whitethroat | **-0.15** | **<0.001** | Marsh warbler | -0.05 | 0.44 | House sparrow | -0.03 | 0.60 |
| Great tit | **-0.14** | **<0.001** | Garden warbler | -0.03 | 0.53 | Great tit | -0.02 | 0.68 |
| House sparrow | **-0.13** | **<0.001** | Willow warbler | -0.03 | 0.67 | Common nightingale | 0.00 | 0.96 |
| Blue tit | **-0.13** | **<0.001** | Chaffinch | -0.02 | 0.66 | Wren | 0.00 | 0.99 |
| Garden warbler | **-0.12** | **<0.001** | Yellowhammer | -0.02 | 0.84 | Reed bunting | 0.00 | 0.97 |
| Long-tailed tit | **-0.12** | **<0.001** | Lesser whitethroat | 0.00 | 0.99 | Dunnock | 0.03 | 0.50 |
| Blackcap | **-0.12** | **<0.001** | Blackbird | 0.01 | 0.84 | Greenfinch | 0.04 | 0.48 |
| Chaffinch | **-0.11** | **<0.001** | Reed warbler | 0.01 | 0.88 | Song thrush | 0.04 | 0.64 |
| Dunnock | **-0.11** | **<0.001** | Sedge warbler | 0.05 | 0.50 | Marsh warbler | 0.05 | 0.47 |
| Bullfinch | **-0.06** | **<0.001** | Bullfinch | 0.06 | 0.18 | Goldfinch | 0.08 | 0.14 |
| Greenfinch | **-0.06** | **0.01** | Goldfinch | 0.07 | 0.10 | Robin | 0.08 | 0.25 |
| Yellowhammer | -0.05 | 0.37 | Song thrush | 0.09 | 0.31 | Whitethroat | 0.09 | 0.24 |
| Robin | -0.03 | 0.23 | Wren | 0.12 | 0.19 | Chiffchaff | 0.09 | 0.09 |

**Table S9:** The species-level significance of synchrony (wavelet phasor mean field magnitudes) in counts, productivity, and survival rates across difference timescales. Red boxes indicate that one or more year has significant synchrony varying at that timescale and green boxes indicate that there was no significant synchrony.

|  | **Timescale** | | | | | | | | | |
| --- | --- | --- | --- | --- | --- | --- | --- | --- | --- | --- |
| **Count** | **2** | **3** | **4** | **5** | | **6** | **7** | **8** | | |
| Blackbird |  |  |  |  | |  |  |  | | |
| Blackcap |  |  |  |  | |  |  |  | | |
| Blue tit |  |  |  |  | |  |  |  | | |
| Bullfinch |  |  |  |  | |  |  |  | | |
| Cettis warbler |  |  |  |  | |  |  |  | | |
| Chaffinch |  |  |  |  | |  |  |  | | |
| Chiffchaff |  |  |  |  | |  |  |  | | |
| Common nightingale |  |  |  |  | |  |  |  | | |
| Dunnock |  |  |  |  | |  |  |  | | |
| Garden warbler |  |  |  |  | |  |  |  | | |
| Great tit |  |  |  |  | |  |  |  | | |
| Lesser whitethroat |  |  |  |  | |  |  |  | | |
| Long-tailed tit |  |  |  |  | |  |  |  | | |
| Reed warbler |  |  |  |  | |  |  |  | | |
| Robin |  |  |  |  | |  |  |  | | |
| Sedge warbler |  |  |  |  | |  |  |  | | |
| Song thrush |  |  |  |  | |  |  |  | | |
| Whitethroat |  |  |  |  | |  |  |  | | |
| Willow warbler |  |  |  |  | |  |  |  | | |
| Wren |  |  |  |  | |  |  |  | | |
| **Productivity** |  | | | | | | | | |  |
| Blackbird |  |  |  | |  |  |  | |  |  |
| Blackcap |  |  |  | |  |  |  | |  |  |
| Blue tit |  |  |  | |  |  |  | |  |  |
| Bullfinch |  |  |  | |  |  |  | |  |  |
| Cettis warbler |  |  |  | |  |  |  | |  |  |
| Chaffinch |  |  |  | |  |  |  | |  |  |
| Chiffchaff |  |  |  | |  |  |  | |  |  |
| Common nightingale |  |  |  | |  |  |  | |  |  |
| Dunnock |  |  |  | |  |  |  | |  |  |
| Garden warbler |  |  |  | |  |  |  | |  |  |
| Great tit |  |  |  | |  |  |  | |  |  |
| Lesser whitethroat |  |  |  | |  |  |  | |  |  |
| Long-tailed tit |  |  |  | |  |  |  | |  |  |
| Reed warbler |  |  |  | |  |  |  | |  |  |
| Robin |  |  |  | |  |  |  | |  |  |
| Sedge warbler |  |  |  | |  |  |  | |  |  |
| Song thrush |  |  |  | |  |  |  | |  |  |
| Whitethroat |  |  |  | |  |  |  | |  |  |
| Willow warbler |  |  |  | |  |  |  | |  |  |
| Wren |  |  |  | |  |  |  | |  |  |
| **Survival rates** |  | | | | | | | | |  |
| Blackbird |  |  |  | |  |  |  | |  |  |
| Blackcap |  |  |  | |  |  |  | |  |  |
| Blue tit |  |  |  | |  |  |  | |  |  |
| Bullfinch |  |  |  | |  |  |  | |  |  |
| Cettis warbler |  |  |  | |  |  |  | |  |  |
| Chaffinch |  |  |  | |  |  |  | |  |  |
| Chiffchaff |  |  |  | |  |  |  | |  |  |
| Common nightingale |  |  |  | |  |  |  | |  |  |
| Dunnock |  |  |  | |  |  |  | |  |  |
| Garden warbler |  |  |  | |  |  |  | |  |  |
| Great tit |  |  |  | |  |  |  | |  |  |
| Lesser whitethroat |  |  |  | |  |  |  | |  |  |
| Long-tailed tit |  |  |  | |  |  |  | |  |  |
| Reed warbler |  |  |  | |  |  |  | |  |  |
| Robin |  |  |  | |  |  |  | |  |  |
| Sedge warbler |  |  |  | |  |  |  | |  |  |
| Song thrush |  |  |  | |  |  |  | |  |  |
| Whitethroat |  |  |  | |  |  |  | |  |  |
| Willow warbler |  |  |  | |  |  |  | |  |  |
| Wren |  |  |  | |  |  |  | |  |  |

*Accounting for transient individuals in survival estimates*

The CJS model makes a number of assumptions which, if violated, may bias parameter estimates (see^1^ for full details). Of particular relevance to the CES dataset is the assumption that every marked individual alive in the population in a given year has the same probability of being recaptured. In the CES dataset this may not be the case, as individuals occupying territories further from the main netting area will have a lower likelihood of being recaught, and individuals migrating through a site in one year are unlikely to be recaught there in subsequent years. These individuals are effectively ‘transient’ in the population and their presence will decrease the average apparent survival probability. We therefore modified the standard CJS model to account for the presence of transient birds by introducing an additional ‘survival period’ in the season of first capture. For each bird we inserted an additional period after the first capture, indicating whether the bird was recaught subsequently in the same season. The probability of surviving this period can be regarded as the probability that the bird is resident on the site (i.e. the probability that it will be available for recapture at the same site in subsequent years). Accounting for transients in these models substantially reduces problems with overdispersion that are typical of capture-recapture data. Residency probability was assumed to be constant between years but allowed to vary between the 10 schemes operating within the Euro-CES because visit number and timing of CES season varies between the schemes (Table S1).

*Variation in synchrony between spatial resolutions*

In 26 species, across Europe, we found that the scale and strength of synchrony is lower at higher resolutions (the scale over which site-level data is pooled) (Figure S2, Table S4). The relative scale (the proportion of species range over which synchrony occurs) is lower at higher resolutions in count synchrony, higher at higher resolutions in productivity synchrony and does not vary with resolution in survival synchrony (Figure S2, Table S4).
